# Machine Learning Nomogram for Predicting Dengue Shock Syndrome in Pediatric Patients With Dengue Fever in Vietnam

**DOI:** 10.7759/cureus.81819

**Published:** 2025-04-07

**Authors:** Rang N Nguyen, Hue T Lam, Hung V Phan, Nghia Q Bui

**Affiliations:** 1 Pediatrics, Can Tho University of Medicine and Pharmacy, Can Tho, VNM; 2 Pediatrics, Bac Lieu General Hospital, Bac Lieu, VNM

**Keywords:** dengue fever, dengue shock syndrome, machine learning, nomogram, vietnam

## Abstract

Background

Early identification of pediatric patients at high risk for dengue shock syndrome (DSS) is crucial to enable timely and appropriate clinical interventions. However, the application of machine learning (ML) models for predicting DSS risk remains underexplored.

Objective

This study aimed to develop and validate a ML-based nomogram for predicting DSS risk in pediatric patients with dengue fever, supporting clinical decision-making.

Methods

A prospective study was conducted on 230 children with dengue fever admitted to Can Tho Children’s Hospital, Vietnam, from January 2020 to December 2022. Clinical and laboratory data were collected and analyzed using R software (version 4.4.1). Six ML algorithms were used to develop risk prediction models for hospitalized pediatric patients with dengue, and their predictive performances were compared. The best-performing model was used to construct a nomogram for DSS prediction. Model performance was evaluated using the area under the receiver operating characteristic curve (AUROC), and the calibration of the nomogram was assessed using a calibration curve.

Results

Among the 230 dengue patients enrolled, 124 (53.9%) were male, with a median age of 11 years (IQR: 8-13 years). The cohort was randomly divided into a training set (n = 173) and a test set (n = 57). Five key predictors selected for the nomogram were albumin, activated partial thromboplastin time (APTT), fibrinogen, aspartate aminotransferase (AST), and platelet count. In the test set, the AUROC for the six models ranged from 0.888 to 0.945. The random forest model demonstrated the best performance, with an AUROC of 0.945 (95% CI: 0.886-1.000), an accuracy of 0.951 (95% CI: 0.865-0.989), sensitivity of 0.894, specificity of 0.976, and a Kappa score of 0.884.

Conclusions

ML-based models can be established and potentially help identify hospitalized pediatric patients with dengue who are at high risk of progressing to DSS. The proposed nomogram may be a valuable tool for predicting DSS in clinical practice.

## Introduction

Dengue fever is a significant global public health concern, particularly in tropical and subtropical regions where Aedes mosquitoes thrive. The dengue virus, a member of the Flavivirus genus, consists of four serotypes (DEN-1, DEN-2, DEN-3, and DEN-4), with DEN-1 and DEN-2 being the most prevalent serotypes circulating in Vietnam [1,2]. The WHO estimates that approximately 390 million dengue infections occur annually, with around 96 million resulting in symptomatic illness. Severe forms of the disease, including dengue hemorrhagic fever (DHF) and dengue shock syndrome (DSS), contribute to an estimated 10,000-20,000 deaths each year, primarily among children and young adults. The global incidence of dengue in children and adolescents surged from 13.2 million cases in 1990 to 21.6 million cases in 2021, representing a 64.4% rise [3,4].

Vietnam has one of the highest burdens of dengue in Southeast Asia, with recurrent outbreaks reported each year. Between 2000 and 2015, the country recorded 847,929 dengue cases, translating to an annual incidence rate of 168 cases per 100,000 population [5]. While most cases present as mild or moderate illness, approximately 5% progress to severe dengue or DSS, conditions associated with high morbidity and mortality if not promptly managed [6]. The disease typically progresses from an initial febrile phase to a critical phase around days 4 to 6 of illness, during which plasma leakage, severe bleeding, and organ impairment may develop [7]. Rapid identification of patients at high risk for DSS is crucial to initiate timely interventions, optimize resource allocation, and improve clinical outcomes.

Current methods for predicting DSS rely primarily on traditional statistical approaches such as univariate and multivariate logistic regression models. While these methods have contributed to understanding risk factors, they are often limited in their sensitivity, specificity, and predictive accuracy [8-15]. Machine learning (ML), an emerging field of artificial intelligence, has revolutionized predictive modeling in medicine by analyzing large datasets and identifying complex patterns beyond the capacity of traditional statistical techniques. ML algorithms have demonstrated superior predictive performance across various medical domains, including infectious disease prognosis and critical care decision-making [16].

Prior ML studies on DSS prediction have predominantly focused on outpatient cohorts, utilizing a restricted set of clinical variables. This limitation may compromise the generalizability of these models to hospitalized pediatric populations, who often present with more complex clinical profiles. ML offers a potential solution by enabling the development of more sophisticated models that can capture these non-linear relationships and improve prediction accuracy. This enhanced predictive capability is particularly valuable in the creation of nomograms for the prediction of DSS. Despite the potential benefits, ML is not widely used for predicting DSS, particularly in Vietnamese children.

This study aims to bridge this gap by developing and validating an ML-driven risk prediction model for DSS in hospitalized children with dengue. By leveraging ML algorithms, we seek to improve the accuracy of DSS prediction and develop a clinically applicable risk prediction nomogram. This tool has the potential to aid clinicians in identifying high-risk patients early in the disease course, enabling timely and targeted management strategies to reduce dengue-related complications and mortality.

## Materials and methods

Study population and design

A prospective cohort study was conducted from January 2020 to December 2022 at Can Tho Children's Hospital, Vietnam. The study enrolled 230 Vietnamese children with confirmed dengue fever. Written informed consent was obtained from the guardians of all participants, and the study was conducted in accordance with the Declaration of Helsinki.

Inclusion and exclusion criteria

The diagnosis of dengue fever was confirmed using a Rapid Test for Dengue NS1 Ag (SD Bioline) and enzyme-linked immunosorbent assay (ELISA) for dengue virus IgM. All children with laboratory-confirmed dengue infection were eligible for inclusion. Patients with pre-existing hematological disorders, including anemia, leukemia, idiopathic thrombocytopenic purpura, or sepsis, were excluded to avoid potential confounding effects on laboratory parameters.

Data collection

Baseline data were collected upon admission and included both demographic and laboratory parameters: age, sex, weight, height, complete blood count (including WBC count, hematocrit, and platelet count), liver enzymes (aspartate aminotransferase (AST) and alanine aminotransferase (ALT)), albumin, urea, creatinine, coagulation profiles (activated partial thromboplastin time (APTT), prothrombin time (PT), and fibrinogen), glycemia, and electrolyte levels (sodium, potassium, chloride).

The complete blood counts were performed using the Siemens Advia 2120i (Austria), biochemical assays were conducted on the Beckman Coulter AU 480 (USA), and coagulation parameters were measured using the Sysmex CS 200i (Japan).

Operational definitions

Target Variable

DSS is defined as severe plasma leakage leading to shock, characterized by tachycardia, weak pulse, narrowing of the pulse pressure (a difference between diastolic and systolic pressures ≤20 mmHg), delayed capillary refill, or hypotension accompanied by cold, clammy skin and restlessness [7].

Candidate Features

Fifteen candidate variables were selected based on previous literature. These variables were dichotomized using clinically relevant thresholds to distinguish between uncomplicated dengue fever and DSS. The variables included: age: ≤5 years vs. >5 years; overweight/obesity: BMI above the 85th percentile; hematocrit: ≥ 20% above the age-specific average; WBC count: ≤4 × 10⁹/L; platelet count: ≤50 × 10⁹/L; AST: ≥ 120 U/L; ALT: ≥ 120 U/L; APTT: Prolonged (≥ 20% above the control value); PT: Prolonged (≥ 3 seconds above the control value); fibrinogen: ≤150 mg/dL (indicative of hypofibrinogenemia); sodium: ≤ 130 mmol/L (hyponatremia); creatinine: ≥ 1.1 mg/dL; glucose: ≤ 70 mg/dL (hypoglycemia) [8,9,12-14,17].

Model development and validation

The 230 dengue patients were randomly assigned into a training set (75%) and a test set (25%). Six ML algorithms were employed to develop predictive models for DSS: Random Forest (RF); Support Vector Machine (SVM); Decision Tree (DT); Artificial Neural Network (ANN); Naïve Bayes (NB); K-Nearest Neighbors (KNN). Each model was optimized using three repetitions of 10-fold cross-validation or parameter tuning. Hyperparameter tuning was performed for each specific ML model as follows: RF (mtry=4, min.node.size=1, ntree=500); SVM (sigma=0.1, C=10); DT (cp=0.01, maxdepth=5, minsplit=20); ANN (size=5, decay=0.01); NB (userkernel=TRUE, adjust=1, fL=1); and KNN (k=5). The performance of the models was evaluated in both the training and test sets using the following metrics: area under the receiver operating characteristic curve (AUROC); Accuracy; Sensitivity; Specificity; Positive Predictive Value (PPV) and Negative Predictive Value (NPV); Kappa Score. The optimal ML algorithm was selected based on these performance metrics. Subsequently, the most significant risk factors identified by the optimal model were used to construct a nomogram. The nomogram’s discrimination and calibration were assessed using a calibration curve.

Ethical approval

This study received ethical approval from the Ethics Committee in Biological Research of Can Tho University of Medicine and Pharmacy (Registration No: 734-QD-DHYDCT). All procedures adhered to ethical guidelines for research involving human subjects.

Statistical analysis

Categorical variables are presented as counts and percentages, whereas continuous variables are summarized as medians with IQRs. The Chi-squared test was used to compare categorical variables, and the Mann-Whitney U test was applied for continuous variables. Statistical analyses and data visualization were performed using R version 4.4.1 (R Foundation for Statistical Computing, Vienna, Austria). Key R packages utilized included caret, randomForest, rpart, e1071, neuralnet, and pROC for model fitting and performance evaluation, and the rms package for nomogram construction and calibration. A p-value < 0.05 was considered statistically significant.

## Results

A total of 230 pediatric patients diagnosed with dengue fever were enrolled in this study. The median age was 11 years (IQR: 8-13 years), and 124 patients (53.9%) were male. Among the study population, 69 children developed DSS, while 161 were classified as having non-severe DF.

Baseline demographic and laboratory characteristics are summarized in Table 1. Significant differences were observed between the DSS and DF groups in several parameters, including HCT, platelet count, AST, ALT, APTT, PT, fibrinogen levels, and plasma sodium (all p < 0.001). In addition, hypoglycemia and age also differed significantly between the two groups (p < 0.05). No significant differences were detected for sex, overweight/obesity, WBC count, and creatinine levels (p > 0.05).

**Table 1:** The laboratory test results and demographic characteristics at baseline. DSS: Dengue shock syndrome; DF: Dengue fever; HCT*: Hematocrit 20% average for age; AST: Aspartate aminotransferase; ALT: Alanine aminotransferase; APTT: Activated partial thromboplastin time; PT: Prothrombin time.

Variables	Total (n=230)	DSS (n=69)	DF (n=161)	P-value
Number, n (%)	230 (100.0)	69 (30.0)	161 (70.0)	
Gender				
Male	124 (53.9)	33 (47.8)	91 (56.5)	0.225
Female	106 (46.1)	36 (52.2)	70 (43.5)	
Age, years				
1-5	45 (19.6)	8 (11.6)	37 (23.0)	0.046
6-15	185 (80.4)	61 (88.4)	124 (77.0)	
Overweight/Obesity				
Yes	45 (19.6)	15 (21.7)	30 (18.6)	0.586
No	185 (80.4)	54 (78.3)	131 (81.4)	
WBC ≤ 4x 10^9^/L				
Yes	106 (46.1)	26 (37.7)	80 (49.7)	0.094
No	124 (53.9)	43 (62.3)	81 (50.3)	
HCT*				
Yes	115 (50.0)	53 (76.8)	62 (38.5)	<0.001
No	115 (50.0)	16 (23.2)	99 (61.5)	
Platelet ≤ 50 x 10^9^/L				
Yes	102 (44.3)	64 (92.8)	38 (23.6)	<0.001
No	128 (55.7)	5 (7.2)	123 (76.4)	
Albumin ≤35 g/L				
Yes	76 (33.0)	65 (94.2)	11 (6.8)	< 0.001
No	154 (67.0)	4 (5.8)	150 (93.2)	
AST ≥120 U/I				
Yes	57 (24.8)	46 (66.7)	11 (6.8)	<0.001
No	173 (75.2)	23 (33.3)	150 (93.2)	
ALT ≥120 U/I				
Yes	25 (10.9)	20 (29.0)	5 (3.1)	<0.001
No	205 (89.1)	49 (71.0)	156 (96.9)	
APTT ≥40 sec				
Yes	68 (29.6)	12 (17.4)	150 (93.2)	<0.001
No	162 (70.4)	57 (82.6)	11 (6.9)	
PT ≥15 sec				
Yes	15 (6.5)	14 (20.3)	1 (0.6)	<0.001
No	215 (93.5)	55 (79.7)	160 (99.4)	
Fibrinogen ≤150 mg/dL				
Yes	43 (18.7)	40 (58.0)	3 (1.9)	<0.001
No	187 (81.3)	29 (42.0)	158 (98.1)	
Glycemia ≤70 mg/dL				
Yes	10 (95.7)	6 (8.7)	4 (2.5)	0.034
No	220 (4.3)	63 (91.3)	157 (97.5)	
Creatinine ≥1.1 mg/dL				
Yes	13 (5.7)	7 (10.1)	6 (3.7)	0.053
No	217 (94.3)	62 (89.9)	155 (96.3)	
Sodium ≤130 mmol/L				
Yes	13 (5.7)	11 (15.9)	2 (1.2)	<0.001
No	217 (94.3)	58 (84.1)	159 (98.8)	

Development of ML models

To develop ML models for DSS prediction, the dataset was randomly split into a training set (n = 173, 75%) and a test set (n = 57, 25%). Among the training set, 53 patients (30.6%) developed DSS, while the test set included 16 DSS cases (28.1%). The ML models were developed using a range of demographic and laboratory variables, including gender, age, overweight/obesity status, WBC, HCT, platelet count, AST, ALT, APTT, PT, fibrinogen, albumin, glucose, creatinine, and plasma sodium. Model performance metrics, including AUROC, accuracy, sensitivity, specificity, positive predictive value (PPV), and negative predictive value (NPV), are presented. The comparative performance of all six ML algorithms in both the training and test sets is shown in Table 2.

**Table 2:** Predictive performance comparison of the six types of machine learning algorithms. Sen: Sensitivity; Spec: Specificity; PPV: Positive predictive value; NPV: Negative predictive value; MC: Machine learning; AUROC: Area under receiver operating curve; SVM: Support vector machine.

	AUROC	Accuracy	Sen	Spec	PPV	NPV	Kappa
Test set							
Random forest	0.945 (0.886, 1.000)	0.951 (0.865, 0.989)	0.894	0.976	0.944	0.954	0.884
Decision tree	0.921 (0.840, 1.000)	0.935 (0.843, 0.982)	0.888	0.954	0.888	0.954	0.843
K-nearest neighbors	0.905 (0.814, 0.996)	0.935 (0.843, 0.982)	0.935	0.934	0.833	0.977	0.838
Support vector machine	0.938 (0.871, 1.000)	0.935 (0.843, 0.982)	0.850	0.976	0.944	0.931	0.848
Neural network	0.921 (0.840, 1.000)	0.935 (0.843, 0.982)	0.888	0.954	0.888	0.954	0.843
Naïve Bayes	0.888 (0.790-0.987)	0.935 (0.843, 0.982)	1.000	0.916	0.777	1.000	0.832
Training set							
Random forest	0.985 (0.965, 1.000)	0.988 (0.957, 0.998)	0.980	0.991	0.980	0.991	0.971
Decision tree	0.941 (0.905, 0.978)	0.934 (0.885, 0.966)	0.844	0.981	0.960	0.923	0.850
K-nearest neighbors	0.894 (0.839, 0.950)	0.922 (0.871, 0.958)	0.913	0.926	0.823	0.965	0.811
Support vector machine	0.915 (0.866, 0.964)	0.928 (0.878, 0.962)	0.882	0.948	0.882	0.948	0.831
Neural network	0.959 (0.926, 0.991)	0.958 (0.916, 0.983)	0.907	0.982	0.960	0.957	0.903
Naïve Bayes	0.868 (0.807, 0.929)	0.916 (0.864, 0.953)	0.974	0.899	0.745	0.991	0.788

**Figure 1:**
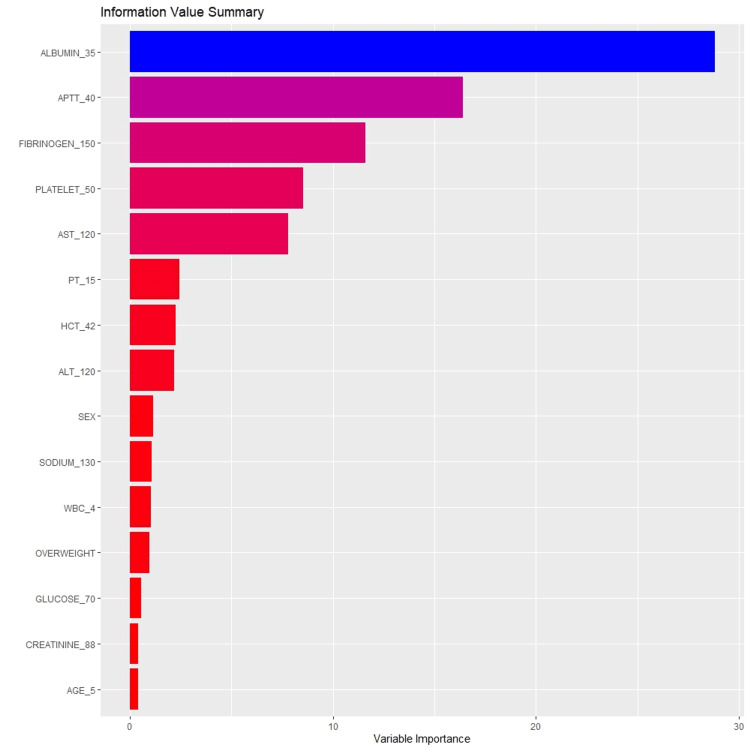
The fifteen important features were derived from the random forest algorithm.

In the training set, the random forest algorithm achieved the highest performance with an AUROC of 0.985, accuracy of 0.988, sensitivity of 0.980, specificity of 0.991, PPV of 0.980, and NPV of 0.991. In the test set, the random forest model also outperformed the other algorithms, achieving an AUROC of 0.945 (95% CI: 0.886-1.000), accuracy of 0.951 (95% CI: 0.865-0.989), sensitivity of 0.894, specificity of 0.976, PPV of 0.944, NPV of 0.954, and a Kappa score of 0.884. The important features of the random forest model are presented in Figure 1.

Feature selection and nomogram development

**Figure 2:**
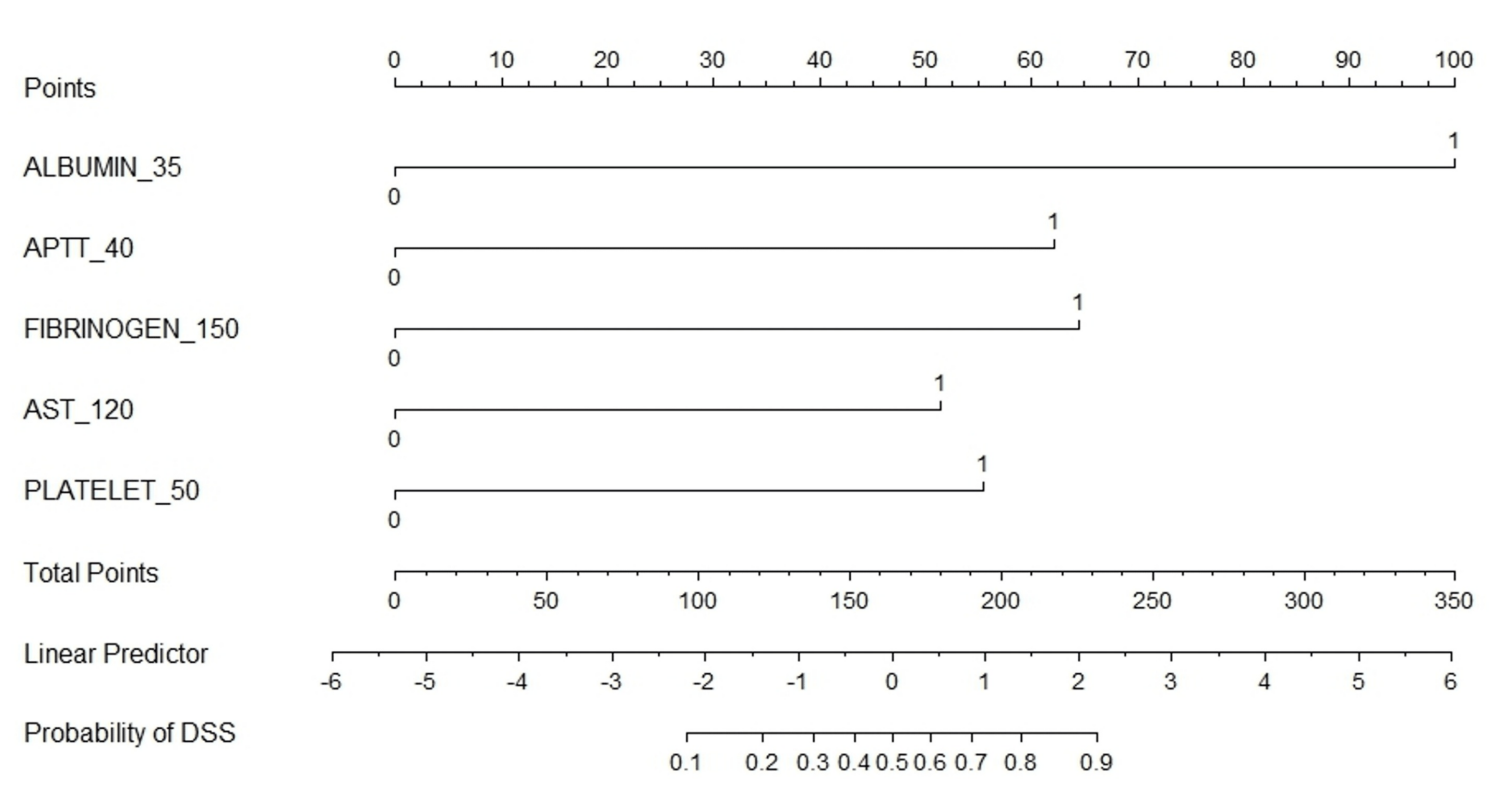
A nomogram for estimating the risk of dengue shock syndrome.

The 5 most critical features (albumin, APTT, fibrinogen, AST, and platelet count) were incorporated into a visual nomogram for estimating the risk of DSS in pediatric dengue patients (Figure 2). The nomogram provides a user-friendly tool for clinicians to estimate DSS risk by summing the points associated with each patient’s laboratory values. For example, a patient with albumin ≤ 35 g/L (100 points), APTT ≥ 40 seconds (62 points), AST ≥ 120 U/L (50 points), and platelet count ≤ 50 x 10⁹/L (55 points) would have a total score of 267, corresponding to an estimated DSS risk of 96%. Adding fibrinogen ≤ 150 mg/dL (65 points) would result in an almost 100% predicted risk of DSS.

Nomogram‘s calibration

**Figure 3:**
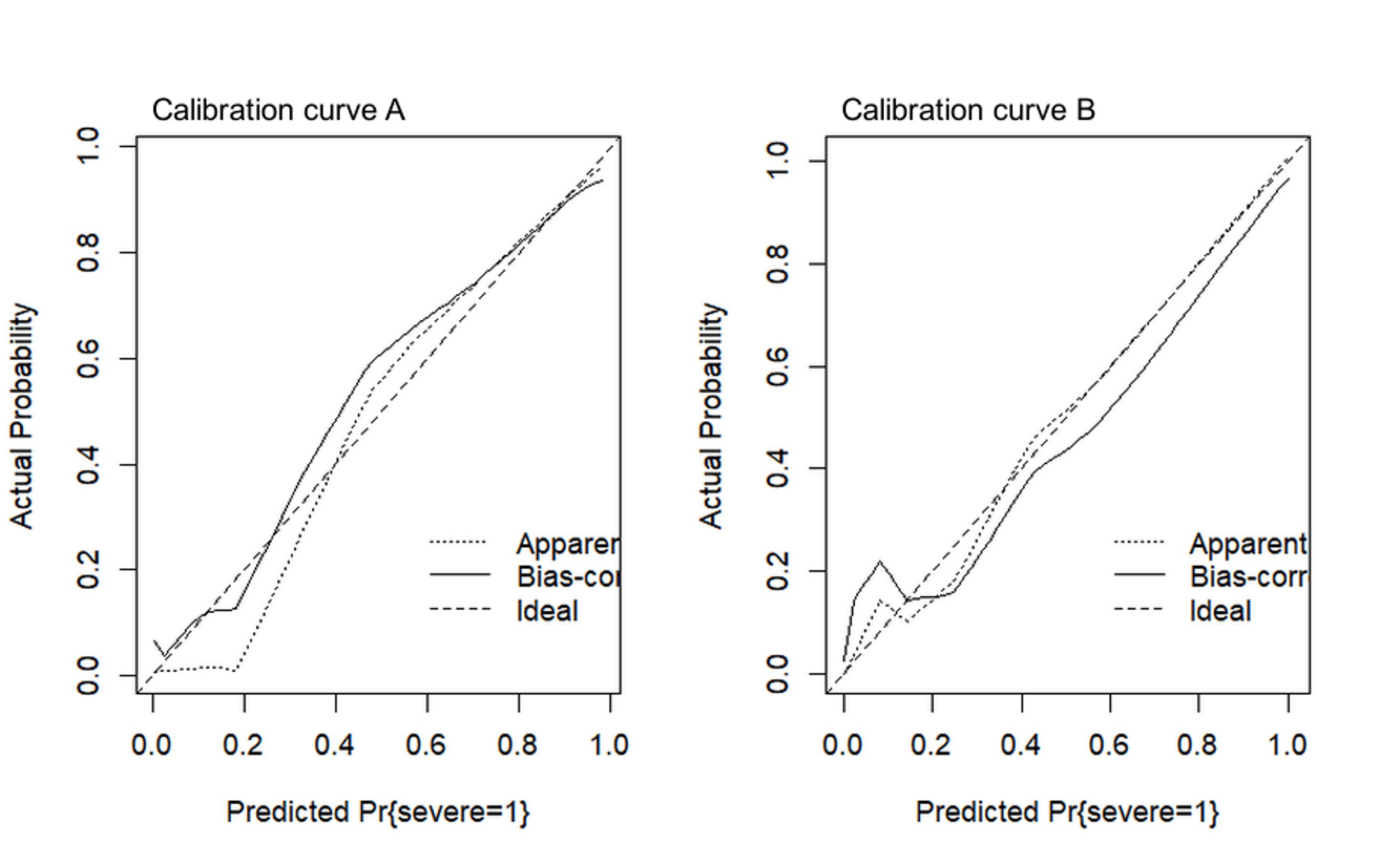
Nomogram calibration curve for the training set (A) and test set (B). The X-axis represents the predicted probability of DSS for pediatric patients, and the Y-axis represents the actual probability. A model's calibration was determined by the degree of curve alignment with the diagonal.

The nomogram’s calibration was evaluated using calibration curves in both the training set and the test set. The calibration plots demonstrated strong agreement between predicted and observed probabilities of DSS, with only minor deviations from the ideal line. The mean absolute error was 0.048 in the training set (calibration curve A) and 0.045 in the test set (calibration curve B), indicating that the nomogram exhibited excellent calibration and predictive reliability (Figure 3).

## Discussion

This study aimed to develop a nomogram for predicting the risk of shock in pediatric dengue patients using explainable ML. This nomogram, using routine lab values (albumin, APTT, fibrinogen, AST, platelets), allows automated DSS risk assessment in electronic medical records. Immediate risk scores and alerts support clinicians' point-of-care decisions at hospital admission, particularly in resource-limited settings.

While previous research has identified risk factors for DSS through traditional statistical methods [11-14, 18-22], our study leverages advanced ML techniques to improve predictive accuracy. A comprehensive meta-analysis by Huy NT et al. highlighted key risk factors for DSS, including demographic characteristics (age, female gender), clinical symptoms (neurological manifestations, nausea/vomiting, abdominal pain, gastrointestinal bleeding), laboratory findings (hemoconcentration, hypoalbuminemia, hypoproteinemia, altered liver enzymes, coagulation abnormalities), and virological factors (infection type and virus serotype) [22].

In our study, we selected 15 DSS-related risk factors, including age, sex, overweight status, white blood cell, hematocrit, platelet count, albumin, APTT, PT, fibrinogen, AST, ALT, creatinine, blood glucose, and plasma sodium. Among ML algorithms, the random forest model demonstrated the highest predictive accuracy. The model performed well in both training and validation sets, with an AUROC of 0.985 (95% CI: 0.965-1.000) and accuracy of 0.988 (95% CI: 0.957-0.998) in the training set, and an AUROC of 0.945 (95% CI: 0.886-1.000) with an accuracy of 0.951 (95% CI: 0.865-0.989) in the validation set. This model exhibited superior predictive performance compared to Tran PN et al.'s prior study, which pooled two observational cohorts of 4,522 dengue patients and developed an ML-based model incorporating five features (body weight, vomiting history, liver size, HCT, platelet count) with an AUROC of 0.85 (95% CI: 0.81-0.90) [23]. Similarly, a study on 7,563 Vietnamese children with ≤3 days of fever developed a nomogram using four features (vomiting, platelet count, AST, and a positive NS1 test), achieving an AUROC of 0.95, sensitivity of 87% (95% CI: 80%-92%), and specificity of 88% (95% CI: 87%-89%) [24].

Unlike these outpatient-focused studies, our research targeted hospitalized dengue patients. In addition to thrombocytopenia and elevated AST, our findings identified hypoalbuminemia and coagulation disorders, including prolonged APTT and hypofibrinogenemia, as significant predictors of DSS. The five most crucial predictive factors in our model were albumin, APTT, fibrinogen, AST, and platelet count.

Hypoalbuminemia (albumin ≤ 35 g/L) was identified as the strongest predictor of DSS. This finding is consistent with previous studies. In a cohort of 330 Vietnamese children with dengue, an albumin level ≤ 35 g/L was associated with an increased risk of shock within three days (OR = 3.3; 95% CI: 1.8-6) [9]. Similarly, Aung MT et al. reported that serum albumin ≤ 35 g/L was a significant predictor of severe dengue in a study of 144 Myanmar patients (OR = 8.10; 95% CI: 2.55-25.72; p < 0.001) [25]. A meta-analysis of 17 studies further reinforced the link between hypoalbuminemia and severe dengue, showing a relative risk of 2.286 (95% CI: 1.308-3.996) [26].

Prolonged APTT and hypofibrinogenemia were also strongly associated with DSS. In a study of 167 Vietnamese children with dengue, Wills B et al. observed early-stage thrombocytopenia, prolonged APTT, and hypofibrinogenemia [27]. Similarly, an analysis of 471 hospitalized dengue patients in Paraguay identified prolonged APTT (OR = 4.0; 95% CI: 1.6-10, p < 0.001) and low fibrinogen (OR = 2.5; 95% CI: 1-5.9, p = 0.02) as predictors of DSS [19]. However, our study found that prolonged PT did not contribute to DSS risk, consistent with findings from a Malaysian cohort of 732 children with dengue [28].

Thrombocytopenia was another key risk factor for DSS. Prior research has established a strong correlation between DSS and low platelet counts, emphasizing the importance of daily platelet monitoring in dengue management [11, 24]. A meta-analysis by Yuan K et al. reinforced this association, showing a significant relationship between thrombocytopenia and severe dengue (OR = 8.146, 95% CI: 3.374-19.665, p < 0.001) [15].

Liver dysfunction has been reported at high rates in Asian dengue patients, with AST/ALT levels frequently elevated in severe cases [29]. On days 4-6 of illness, Huy BV and Toàn NV found that pediatric patients with AST > 400 U/L were more likely to develop severe dengue (OR = 3.0; 95% CI: 1.1-7.9) [9]. A Malaysian study further demonstrated that the AST²/ALT composite index was the most accurate predictor of severe dengue, with an AUC of 0.83 (95% CI: 0.73-0.93) [30]. These findings align with our results, reinforcing the association between elevated AST and DSS.

This study has several strengths, including its prospective design, comprehensive laboratory assessments before shock onset, and a predictive model with high discrimination and calibration. However, some limitations should be acknowledged. First, the small sample size, single-center nature, and lack of external validation suggest that further research is needed to confirm the generalizability of our results. Second, the conversion of continuous variables into binary categories may have reduced model performance due to information loss. Third, clinical variables such as vomiting, abdominal pain, and hepatomegaly were excluded due to their subjective nature. Finally, we did not differentiate between dengue virus serotypes or primary versus secondary infections. In future research, we will collect data to obtain a larger sample size, combine it with clinical signs, use continuous variables, and perform external validation to improve the model's ability to predict DSS in hospitalized dengue patients.

## Conclusions

In conclusion, this study demonstrates the significant potential of ML to improve the prediction of DSS in pediatric hospital settings. A clinically applicable nomogram, derived from readily available laboratory data, was developed and validated. This tool, incorporating albumin, APTT, fibrinogen, AST, and platelet count, exhibits robust discrimination and calibration, offering valuable clinical guidance for early DSS risk stratification in tropical pediatric populations.

Applying ML to predict dengue severity raises concerns about data privacy, algorithmic bias, and transparency. We adhere to relevant data privacy regulations and local laws. Additionally, ML models should be transparent and easy to understand, serving as a tool to support doctors rather than replace their expertise. This ensures trust and reliability in AI-driven healthcare.
